# A Five-Region Hypothesis Test for Exposure-Disease Associations

**DOI:** 10.1038/s41598-017-05301-4

**Published:** 2017-07-11

**Authors:** Han-Yi Shih, Wen-Chung Lee

**Affiliations:** 1 Institute of Epidemiology and Preventive Medicine, College of Public Health, National Taiwan University, Taipei, Taiwan; 2 Research Center for Genes, Environment and Human Health, College of Public Health, National Taiwan University, Taipei, Taiwan

## Abstract

Characterizing exposure-disease associations is a central issue in epidemiology, one which epidemiologists often approach by adopting the index of the odds ratio and presenting its point estimate, p-value and confidence interval. In this study, the parameter space of the odds ratio is partitioned into five mutually exclusive regions corresponding to ‘strong protective factor’, ‘weak protective factor’, ‘no association’, ‘weak risk factor’, and ‘strong risk factor’, respectively. The authors presented a suite of statistical methods tailored to such a five-region demarcation, including methods for hypothesis testing, confidence interval estimation and calculation of the sample size needed to obtain the desired level of statistical power. The authors show that the five-region methods can efficiently and informatively describe a putative exposure-disease association, including its presence or absence, as well as its direction and strength (if any association exists). Three published results were re-analyzed to demonstrate the methods. R code is provided for convenience as well. The five-region methods are recommended for routine use during the analysis of epidemiologic data.

## Introduction

Characterizing exposure-disease associations is a central issue in epidemiology. Epidemiologists often approach this issue by adopting the index of the odds ratio; presenting its point estimate, p-value and confidence interval; then declaring an exposure a risk factor for the disease if its odds ratio is statistically significantly greater than 1.0, or a protective factor if it is statistically significantly less than 1.0^1^.

Identifying a risk or protective factor for the disease may not be enough. We may also want to determine the strength of such an exposure-disease association. For example, we may want to know whether the odds ratio is statistically significantly greater than 2.0, indicating a strong risk factor; or between 0.5 and 1.0, indicating a weak protective factor. Conventional hypothesis testing for the odds ratio is for determining whether it is 1.0 or not: {H_0_: OR = 1; H_1_: OR < 1 or OR > 1}, so can only be used to infer whether the exposure under study is a risk or protective factor for the disease. Further testing with regards to the cut-off points, such as {H_0_: OR ≥ 0.5; H_1_: OR < 0.5} or {H_0_: OR ≤ 2; H_1_: OR > 2}, incurs a multiple-testing penalty, and therefore, a power loss.

Goeman *et al*.^2^ proposed ‘three-sided testing’, a testing framework for simultaneous testing of ‘inferiority’, ‘equivalence’ and ‘superiority’ in clinical trials. A cardinal feature of Goeman *et al*.’s method is that the three hypotheses can be simultaneously performed at a significance level of *α*, while the family-wise error rate remains within *α*. The approach was later adopted outside of clinical trial settings to compare two digital communication systems^3^. However, our intended, more informative characterization of an exposure-disease association requires partitioning the parameter space of the odds ratio into a total of *five* ‘regions’, which is more than the *three* ‘sides’ that Goeman *et al*.^2^ had considered.

In this paper, we expand the applicability of Goeman *et al*.’s three-sided methods^2^ to the five-region characterization of exposure-disease associations. Our methods (including hypothesis testing, confidence interval estimation, and sample size calculation) are a reinterpretation of the results of the three-sided testing in terms of five regions. We will show that the ‘five-region methods’ are more statistically efficient than traditional methods in describing exposure-disease associations. Data from three published studies are re-analyzed to demonstrate the methods.

## Methods

### Five-Region Demarcation and the Partitioning Principle

Assuming that two cut-off points for the odds ratio are chosen: 0.5 and 2. (Proper choices of the cut-off points will be discussed later.) For the lower cut-off point, OR < 0.5 indicates a strong protective factor, and 0.5 ≤ OR < 1 a weak protective factor. For the upper cut-off point, OR > 2 indicates a strong risk factor, and 1 < OR ≤ 2 a weak risk factor. The parameter space of the odds ratio is thus partitioned into five regions: H_I_, H_II_, …, H_V_ as shown below:

1 $$\{\begin{array}{ll}{{\rm{H}}}_{{\rm{I}}}:{\rm{OR}} < 0.5,\, & {\rm{a}}\,{\rm{strong}}\,{\rm{proctective}}\,{\rm{factor}},\\ {{\rm{H}}}_{{\rm{II}}}:0.5\le {\rm{OR}} < 1, & {\rm{a}}\,{\rm{weak}}\,{\rm{proctective}}\,{\rm{factor}},\\ {{\rm{H}}}_{{\rm{III}}}:{\rm{OR}}=1, & {\rm{no}}\,{\rm{association}},\\ {{\rm{H}}}_{{\rm{IV}}}:1 < {\rm{OR}}\le 2, & {\rm{a}}\,{\rm{weak}}\,{\rm{risk}}\,{\rm{factor}},\\ {{\rm{H}}}_{{\rm{V}}}:{\rm{OR}} > 2, & {\rm{a}}\,{\rm{strong}}\,{\rm{risk}}\,{\rm{factor}}.\end{array}$$

Once the five regions have been demarcated, a putative exposure-disease association can be described succinctly and clearly, including its presence or absence, as well as its direction and strength (if any association exists). Five hypothesis tests are to be performed in total, with each checking whether the true odds ratio is within a particular region. The null hypothesis of test H_*i*_ is OR ∈H_*i*_ and the alternative hypothesis is OR ∈ ${{\rm{H}}}_{i}^{{\rm{c}}}$ (the complement of H_*i*_), for *i* = I, II, …, V.

Because the five regions are mutually exclusive, the true value of the odds ratio can lie in at most one of the regions, so that no two hypotheses can be simultaneously true. The ‘partitioning principle’^4^ thus applies here, dictating that all five hypotheses can be simultaneously performed at a level of *α*, while still keeping the family-wise error rate within *α*. See Web Appendix 1 for a proof. Similarly, Shaffer^5^ pointed out that the correction factor for a Bonferroni multiple testing procedure is not the total number of hypotheses tested, but the maximum number of hypotheses that can simultaneously be true. And in our case, the correction factor is 1. In other words, there is no need for multiple testing correction.

### Five-Region Hypothesis Tests

The above partitioning principle says nothing about the types of the tests (two-sided, one-sided, etc). For each hypothesis, any test can therefore be used as long as each of the five tests keeps its respective type I error rate within *α*. Here, we propose to test H_I_ with an *α*-level right-sided test ({H_0_: OR < 0.5; H_1_: OR ≥ 0.5}), H_II_ with one *α*/2-level left-sided test ({H_0_: OR ≥ 0.5; H_1_: OR < 0.5}) and one *α*/2-level right-sided test ({H_0_: OR < 1; H_1_: OR ≥ 1}) simultaneously; H_III_ with an *α*-level two-sided test ({H_0_: OR = 1; H_1_: OR ≠1}), H_IV_ with one *α*/2-level left-sided test ({H_0_: OR > 1; H_1_: OR ≤ 1}) and one *α*/2-level right-sided test ({H_0_: OR ≤ 2; H_1_: OR > 2}) simultaneously; and H_V_ with an *α*-level left-sided test ({H_0_: OR > 2; H_1_: OR ≤ 2}), respectively.

Assuming normality for the log odds ratio, the ‘five-region test’ is detailed below:

2 $$\{\begin{array}{cccc}{\rm{r}}{\rm{e}}{\rm{j}}{\rm{e}}{\rm{c}}{\rm{t}}\,{{\rm{H}}}_{{\rm{I}}}, &  & {\rm{i}}{\rm{f}} & \frac{{\rm{l}}{\rm{o}}{\rm{g}}\,\hat{{\rm{O}}{\rm{R}}}-\,{\rm{l}}{\rm{o}}{\rm{g}}\,0.5}{{\rm{S}}{\rm{E}}}\ge {Z}_{1-\alpha },\\ {\rm{r}}{\rm{e}}{\rm{j}}{\rm{e}}{\rm{c}}{\rm{t}}\,{{\rm{H}}}_{{\rm{I}}{\rm{I}}}, & {\rm{i}}{\rm{f}}\,\,\frac{{\rm{l}}{\rm{o}}{\rm{g}}\,\hat{{\rm{O}}{\rm{R}}}-\,{\rm{l}}{\rm{o}}{\rm{g}}\,0.5}{{\rm{S}}{\rm{E}}}\le {Z}_{\alpha /2} & {\rm{o}}{\rm{r}} & \,\,\frac{{\rm{l}}{\rm{o}}{\rm{g}}\,\hat{{\rm{O}}{\rm{R}}}}{{\rm{S}}{\rm{E}}}\,\ge {Z}_{1-\alpha /2},\\ {\rm{r}}{\rm{e}}{\rm{j}}{\rm{e}}{\rm{c}}{\rm{t}}\,{{\rm{H}}}_{{\rm{I}}{\rm{I}}{\rm{I}}}, &  & {\rm{i}}{\rm{f}} & \,\,\frac{|{\rm{l}}{\rm{o}}{\rm{g}}\,\hat{{\rm{O}}{\rm{R}}}|}{{\rm{S}}{\rm{E}}}\ge {Z}_{1-\alpha /2},\\ {\rm{r}}{\rm{e}}{\rm{j}}{\rm{e}}{\rm{c}}{\rm{t}}\,{{\rm{H}}}_{{\rm{I}}{\rm{V}}}, & {\rm{i}}{\rm{f}}\,\,\frac{{\rm{l}}{\rm{o}}{\rm{g}}\,\hat{{\rm{O}}{\rm{R}}}}{{\rm{S}}{\rm{E}}}\le {Z}_{\alpha /2} & {\rm{o}}{\rm{r}} & \frac{{\rm{l}}{\rm{o}}{\rm{g}}\,\hat{{\rm{O}}{\rm{R}}}-\,{\rm{l}}{\rm{o}}{\rm{g}}\,2}{{\rm{S}}{\rm{E}}}\,\ge {Z}_{1-\alpha /2},\\ {\rm{r}}{\rm{e}}{\rm{j}}{\rm{e}}{\rm{c}}{\rm{t}}\,{{\rm{H}}}_{{\rm{V}}}, &  & {\rm{i}}{\rm{f}} & \frac{{\rm{l}}{\rm{o}}{\rm{g}}\,\hat{{\rm{O}}{\rm{R}}}-\,{\rm{l}}{\rm{o}}{\rm{g}}\,2}{{\rm{S}}{\rm{E}}}\le {Z}_{\alpha },\end{array}$$

where $\widehat{{\rm{OR}}}$ is an estimate of the odds ratio, SE is the standard error of $\mathrm{log}\,\widehat{{\rm{OR}}}$, and *Z*_*x*_ is the *x*’th quantile of the standard normal distribution. As explained earlier, such a five-region test should keep the family-wise error rate within *α*.

Note that the ‘two *α*/2-level one-sided tests simultaneously’ that we used here for H_II_ and H_IV_ are very different from Schuirmann’s procedure for equivalence^6^. For the H_II_ test, the null hypothesis (0.5 ≤ OR < 1) is to be rejected if *either* of the two null hypotheses (OR ≥ 0.5 and OR < 1, respectively) of the ‘*α*/2’-level one-sided tests is rejected. Similarly, for the H_IV_ test, the null hypothesis (1 < OR ≤ 2) is to be rejected if *either* of the two null hypotheses (OR > 1 and OR ≤ 2, respectively) of the ‘*α*/2’-level one-sided tests is rejected. By contrast, in Schuirmann’s procedure, the null hypothesis (nonequivalence) is to be rejected only if *both* null hypotheses (inferiority and superiority, respectively) of the ‘*α*’-level one-sided tests are rejected.

### Five-Region P-Values

Alternatively, one can calculate the ‘five-region p-values’:

3 $$\{\begin{array}{rcl}{{\rm{p}}}_{{\rm{I}}} & = & 1-\varphi (\frac{\mathrm{log}\,\widehat{{\rm{OR}}}-\,\mathrm{log}\,0.5}{{\rm{SE}}}),\\ {{\rm{p}}}_{{\rm{II}}} & = & 2\times \,{\rm{\min }}\{\varphi (\frac{\mathrm{log}\,\widehat{{\rm{OR}}}-\,\mathrm{log}\,0.5}{{\rm{SE}}}),\,1-\varphi (\frac{\mathrm{log}\,\widehat{{\rm{OR}}}}{{\rm{SE}}})\,\},\\ {{\rm{p}}}_{{\rm{III}}} & = & 2\times [1-\varphi (\frac{|\mathrm{log}\,\widehat{{\rm{OR}}}|}{{\rm{SE}}})],\\ {{\rm{p}}}_{{\rm{IV}}} & = & 2\times \,{\rm{\min }}\{\varphi (\frac{\mathrm{log}\,\widehat{{\rm{OR}}}}{{\rm{SE}}}),\,1-\varphi (\frac{\mathrm{log}\,\widehat{{\rm{OR}}}-\,\mathrm{log}\,2}{{\rm{SE}}})\,\},\\ {{\rm{p}}}_{{\rm{V}}} & = & \varphi (\frac{\mathrm{log}\,\widehat{{\rm{OR}}}-\,\mathrm{log}\,2}{{\rm{SE}}}),\end{array}$$

where ϕ(·) is the cumulative distribution function of the standard normal distribution. One then rejects any hypothesis with a corresponding p-value less than or equal to *α*.

Web Appendix 2 shows that for any *i* in {I, II, …, V}, the p_*i*_ in (3) corresponds to the H_*i*_ test in (2), that is, an H_*i*_ will be rejected in (2) if and only if its p_*i*_ ≤ *α* in (3).

### Possible Conclusions in a Five-Region Test

**Figure 1:**
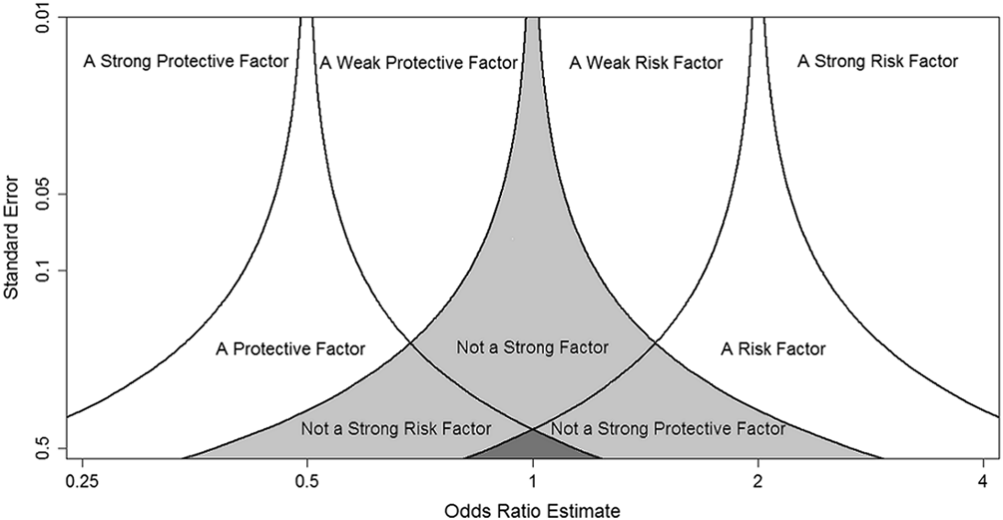
The nine possible conclusions of the five-region test (a strong protective factor: the upper left non-shaded corner; a weak protective factor: the middle left non-shaded area; a protective factor without strength information: the non-shaded area nestled between the upper left non-shaded corner and the middle left non-shaded area; a weak risk factor: the middle right non-shaded area; a strong risk factor: the upper right non-shaded corner; a risk factor without strength information: the non-shaded area nestled between the middle right non-shaded area and the upper right non-shaded corner; not a strong risk factor: the left lightly-shaded area; not a strong protective factor: the right lightly-shaded area; not a strong factor, risk or protective: the middle lightly-shaded area).

If none of the five hypotheses are rejected, a five-region test is inconclusive (represented by the small dark-shaded area in Fig. 1). Otherwise, a conclusion can be made with inferences on the direction and/or the strength of the exposure-disease association; a total of 9 different conclusions (see Fig. 1) are possible in a five-region test, namely, the study exposure is one of the followings:

- (SP) a strong protective factor (the upper left non-shaded corner), if four hypotheses, H_II_, H_III_, H_IV_, and H_V_, are rejected;
- (WP) a weak protective factor (the middle left non-shaded area), if four hypotheses, H_I_, H_III_, H_IV_, and H_V_, are rejected;
- (P) a protective factor without strength information (the non-shaded area nestled between the upper left non-shaded corner and the middle left non-shaded area), if three hypotheses, H_III_, H_IV_, and H_V_, are rejected;
- (WR) a weak risk factor (the middle right non-shaded area), if four hypotheses, H_I_, H_II_, H_III_, and H_V_, are rejected;
- (SR) a strong risk factor (the upper right non-shaded corner), if four hypotheses, H_I_, H_II_, H_III_, and H_IV_, are rejected;
- (R) a risk factor without strength information (the non-shaded area nestled between the middle right non-shaded area and the upper right non-shaded corner), if three hypotheses, H_I_, H_II_, and H_III_, are rejected;
- (NSR) not a strong risk factor (the left lightly-shaded area), if one hypothesis, H_V_, is rejected;
- (NSP) not a strong protective factor (the right lightly-shaded area), if one hypothesis, H_I_, is rejected;
- (NS) not a strong factor, risk or protective (the middle lightly-shaded area), if two hypotheses, H_I_ and H_V_, are rejected.

Note that not all of the total 2^5^ = 32 testing results (a total of five hypotheses tested, each with two possibilities: rejected/not rejected) are possible. For example, it is not possible to have the middle three hypotheses, H_II_, H_III_, and H_IV_, being rejected ($\frac{\mathrm{log}\,\widehat{OR}-\,\mathrm{log}\,0.5}{SE}\le {Z}_{\alpha /2}$ or $\frac{\mathrm{log}\,\widehat{OR}-\,\mathrm{log}\,2}{SE}\,\ge {Z}_{1-\alpha /2}$), while at the same time the remaining two hypotheses, H_I_ and H_V_, both being NOT rejected ($\frac{\mathrm{log}\,\widehat{OR}-\,\mathrm{log}\,0.5}{SE} < {Z}_{1-\alpha }$ and $\frac{\mathrm{log}\,\widehat{OR}-\,\mathrm{log}\,2}{SE} > {Z}_{\alpha }$). If $\frac{\mathrm{log}\,\widehat{OR}-\,\mathrm{log}\,0.5}{SE}\le {Z}_{\alpha /2}$ (H_II_ rejected), we must also have $\frac{\mathrm{log}\,\widehat{OR}}{SE} < {Z}_{\alpha /2}$ (H_III_ and H_IV_ rejected) and $\frac{\mathrm{log}\,\widehat{OR}-\,\mathrm{log}\,2}{SE} < {Z}_{\alpha /2} < {Z}_{\alpha }\,$ (H_V_ rejected), and if $\frac{\mathrm{log}\,\widehat{OR}-\,\mathrm{log}\,2}{SE}\,\ge {Z}_{1-\alpha /2}$ (H_IV_ rejected), we must also have $\frac{\mathrm{log}\,\widehat{OR}}{SE} > {Z}_{1-\alpha /2}\,$ (H_III_ and H_II_ rejected) and $\frac{\mathrm{log}\,\widehat{OR}-\,\mathrm{log}\,0.5}{SE}$ > Z_1−α/2_ > Z_1−α_ (H_I_ rejected).

By comparison, if a classical hypothesis test ({H_0_: OR = 1; H_1_: OR < 1 or OR > 1}) leads to any conclusion at all (i.e., can reject the null hypothesis), it only provides directional information about whether exposure is a protective or a risk factor (represented by the non-shaded region to the left or to the right in Fig. 1: ‘SP+WP+P’ and ‘WR+SR+R’, respectively). When the standard error is relatively small (such as in a study with a relatively large sample size), a five-region test can provide directional information as well as strength information (the upper 4 non-shaded areas in Fig. 1: ‘SP’, ‘WP’, ‘WR’, and ‘SR’, respectively, each requiring the rejections of a total of four hypotheses), while a classical test can only determine the direction. At the other extreme where the standard error is very large (a very small study), a five-region test may still conclusively rule out some remote possibilities for study exposure (represented in the 3 lightly-shaded areas in Fig. 1: ‘NSR’, ‘NSP’, and ‘NS’, respectively), such as that it is not a strong risk factor (H_V_ rejected), not a strong protective factor (H_I_ rejected), or not a strong factor, risk or protective (both H_I_ and H_V_ rejected); at the same *α* level, the classical test leads to none conclusion at all.

Web Appendix 3 presents a function written in R 3.1.2 software (R Foundation for Statistical Computing, Vienna, Austria) code. Input an odds ratio estimate, a standard error, a significance level, and two cut-off points as the arguments of the function, and the function will output the five p-values and the testing results of the five-region test.

### Five-Region Confidence Intervals

The well-known duality between confidence intervals and hypothesis tests dictates that an *α*-level test is to be performed for each and every possible value of the odds ratio, and the resulting non-rejected values constitute a (1−*α*) confidence interval for the odds ratio.

Here, we propose using an *α*-level right-sided test for an odd ratio in H_I_, an *α*-level two-sided test for an odds ratio in {H_II_, H_III_, H_IV_}, and an *α*-level left-sided test for an odds ratio in H_V_. This is essentially Goeman *et al*.’s three-sided testing^2^ (treating H_I_ as inferiority, {H_II_, H_III_, H_IV_} as equivalence, and H_V_ as superiority). Therefore, the resulting ‘five-region confidence interval’ is also identical mathematically to Goeman *et al*.’s three-sided confidence interval^2^.

However, our use of the confidence interval is different from Goeman *et al*.’s. Here, we explicitly check each and every one of the five regions, and reject a region if and only if none of its points fall within the confidence interval. Readers can verify that such use of the confidence interval leads to exactly the same conclusion of a five-region test in the previous section. For an example, see the solid lines (representing the five-region confidence intervals, or equivalently, Goeman *et al*.’s three-sided confidence intervals) and the attached conclusions in a five-region test.

**Figure 2:**
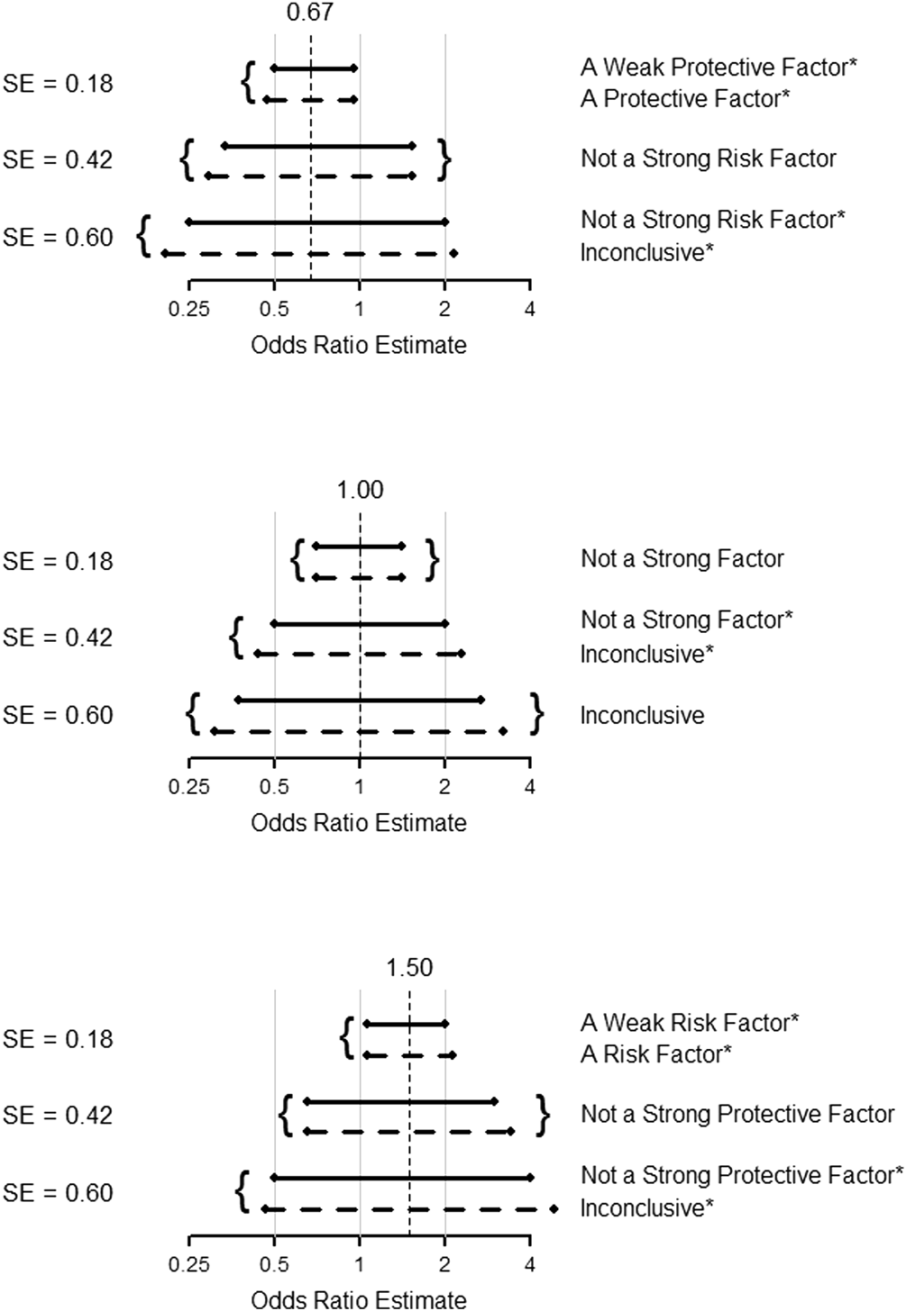
A comparison between the five-region confidence intervals (solid lines) and the classical confidence intervals (dashed lines).

Using the same principle that a region is rejected if none of its point fall within the confidence interval, a classical confidence interval can also be used to determine a five-region conclusion (e.g., see the dashed lines and the attached conclusions in Fig. 2). Figure 2 compares the two approaches (marked ‘*’, if the conclusions are different). The upper panel in Fig. 2 shows the results for$\,\widehat{{\rm{OR}}}=0.67$. When the sample size is small (SE = 0.60, n ≈ 50), the classical confidence interval cannot lead to a conclusion but the five-region confidence interval (or equivalently, the three-sided confidence interval) can rule out the possibility that the exposure is a strong risk factor. With a larger sample size (SE = 0.18, n ≈ 500), the classical confidence interval can only infer a protective factor but the five-region confidence interval (or the three-sided confidence interval, if used to determine a five-region conclusion) can further conclude that its protective effect is weak. The middle panel in Fig. 2 shows the results for$\,\widehat{{\rm{OR}}}=1.00$. With a moderate sample size (SE = 0.42, n ≈ 100), the classical confidence interval cannot reach a conclusion but the five-region confidence interval (or equivalently, the three-sided confidence interval) can rule out a strong factor (either risk or protective). The lower panel in Fig. 2 shows the results for $\,\widehat{{\rm{OR}}}=1.50$. When the sample size is small (SE = 0.60, n ≈ 50), the classical confidence interval cannot reach any conclusions but the five-region confidence interval (or equivalently, the three-sided confidence interval) can rule out a strong protective factor. With a larger sample size (SE = 0.18, n ≈ 500), the classical confidence interval can only infer a risk factor but the five-region confidence interval (or the three-sided confidence interval, if used to determine a five-region conclusion) can further conclude that it is only a weak one.

### Sample Size Calculations

As pointed out earlier, when a five-region test (and its equivalent, a five-region confidence interval, or a three-sided confidence interval used to determine a five-region conclusion) does reach a conclusion it can lead to one of 9 different conclusions. Here we calculate the required sample size to reach a target power of 80% at *α* = 0.05 for each conclusion. (The ‘power’ of a five-region test for a specified conclusion is defined as the probability that the test leads to that specified conclusion or a more precise one.) We performed a total of 100,000 Monte-Carlo simulations to approximate the power for a given sample size and used the bisection method to calculate the sample size needed to reach the target power. For comparison, we used the same method to calculate the sample size needed for a classical confidence interval when used to make a five-region conclusion. Web Appendix 4 presents the R function used.

**Figure 3:**
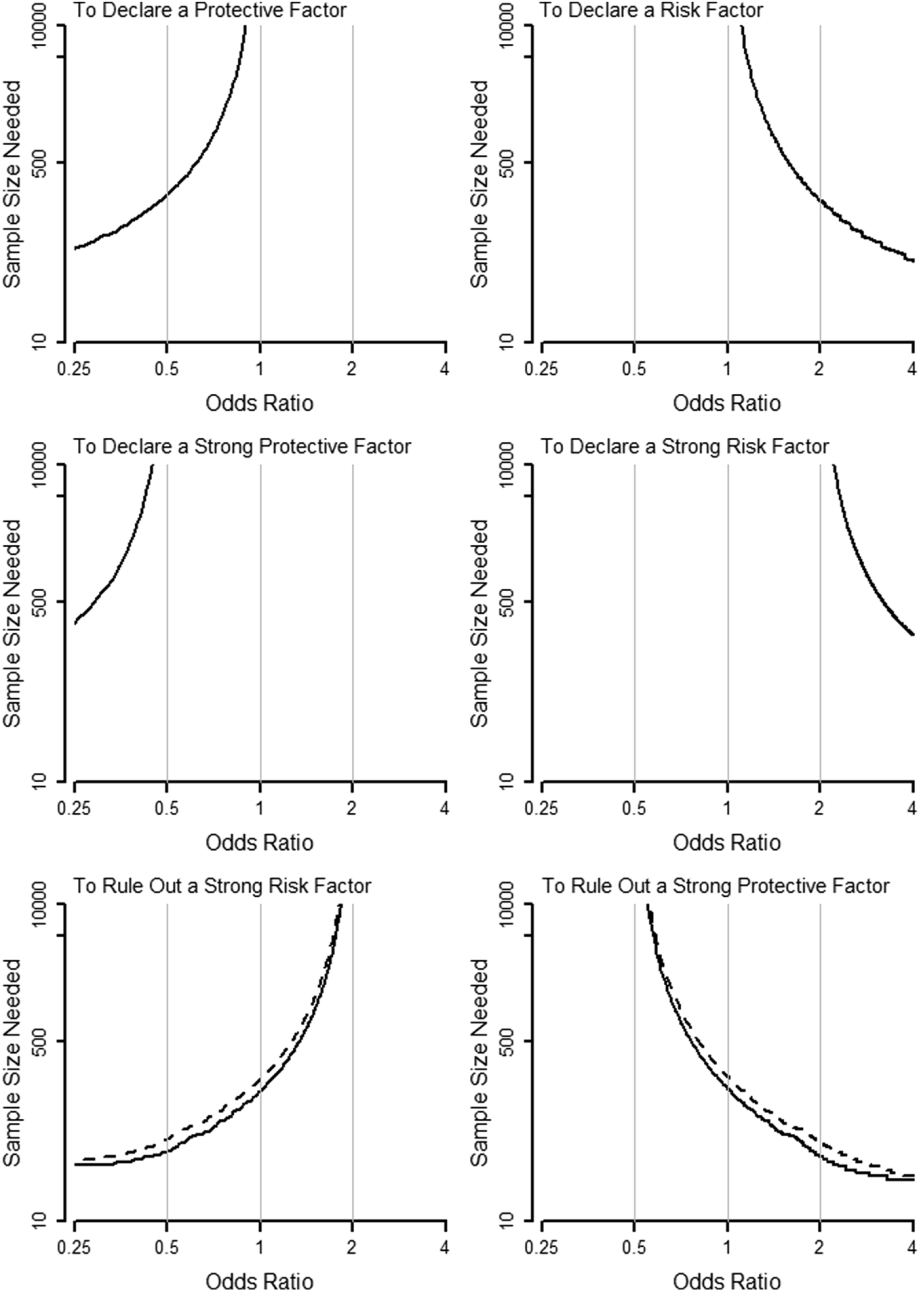
Sample sizes needed for the various conclusions (solid lines: the five-region method; dashed lines: the classical method).

**Figure 4:**
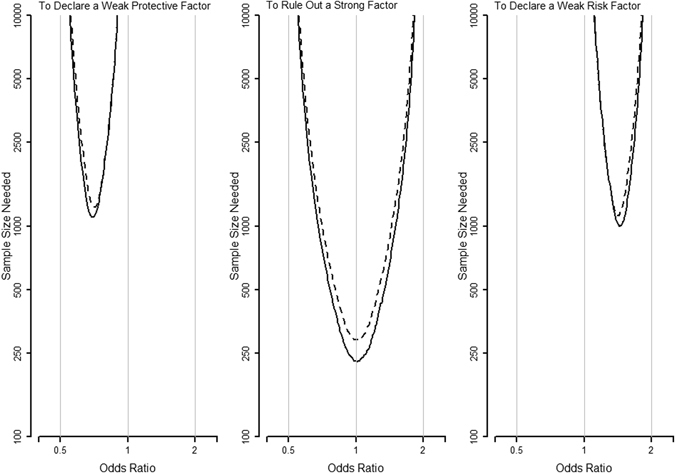
Sample sizes needed for the various conclusions (solid lines: the five-region method; dashed lines: the classical method).

Figures 3 and 4 present the sample sizes (total numbers of subjects) needed for a case-control study with an equal number of cases and controls, conducted in a population with an exposure prevalence of 0.4. To have the required power to reach conclusions for a protective factor, a risk factor, a strong protective factor, and a strong risk factor, respectively (the upper 4 panels in Fig. 3), the required sample sizes are the same for the two methods. For the other 5 conclusions of not a strong risk factor (the lower left panel in Fig. 3), not a strong protective factor (the lower right panel in Fig. 3), a weak protective factor (the left panel in Fig. 4), not a strong factor (the middle panel in Fig. 4), and a weak risk factor (the right panel in Fig. 4), respectively, the required sample sizes for the five-region method (solid lines) are smaller than those of the traditional method (dashed lines).

Figures 3 and 4 also show that the required sample size increases for more precise conclusions as expected. For example, when OR = 1.5, the required sample sizes of the five-region test are 70 (for ruling out a strong protective factor), 642 (for declaring a risk factor), 985 (for ruling out a strong factor), and 1059 (for declaring a weak risk factor), respectively. As another example, when OR = 2.5, the required sample sizes are 32 (for ruling out a strong protective factor), 134 (for declaring a risk factor), and 2198 (for declaring a strong risk factor), respectively.

As many researchers often do not have a very precise aim for their studies at the outset, calculating and comparing the required sample sizes for different conclusions and specific odds ratio values (such as the cases of OR = 1.5 and OR = 2.5 above, if such *a priori* information is available) or for all possible odds ratio values (such as in Figs 3 and 4, if the *a priori* information is lacking) should help researchers better plan their studies under time and cost constraints.

## Examples of Real Data

### Example 1. ‘Not a Strong Risk Factor’ vs. ‘Inconclusive’

Elbaz *et al*.^7^ recruited a total of 196 case-control pairs (matched by sex and age) to examine the association between Parkinson’s disease and preceding nonfatal cancers. A conditional logistic regression was conducted for a matched-data analysis. Elbaz *et al*. reported an adjusted odds ratio of 0.22 [95% (classical) confidence interval: 0.03–2.24] for bladder cancer occurring before the onset of Parkinson’s disease. We calculated a standard error from the confidence interval presented (and likewise for the following two examples). The fact that the value 1 is within the classical confidence interval indicates that the observed negative association between bladder cancer and Parkinson’s disease (OR = 0.22) is not statistically significant. The classical confidence interval itself is also inconclusive since it spans all five regions.

We then used the proposed method to reanalyze the data. The five-region test leads to a rejection (p_V_ = 0.031) of the H_V_ region (a strong risk factor). Similarly, the 95% five-region confidence interval is 0.03–2.00, excluding the H_V_ region entirely. In this example, while neither the classical test nor the classical confidence interval is conclusive about risk of exposure, the five-region test (and the five-region confidence interval) at the same significance level of 0.05 can rule out the possibility that bladder cancer is a strong risk factor for Parkinson’s disease; it can only be one of the following four possibilities: a strong protective factor, a weak protective factor, no association, or a weak risk factor.

### Example 2. ‘Not a Strong Factor, Risk or Protective’ vs. ‘Inconclusive’

Parent *et al*.^8^ recruited a total of 94 melanoma cases and 512 controls to examine the association between night work and risk of cancer among men. Logistic regression was conducted adjusting for a number of potential confounders (age, ancestry, educational level, family income, respondent status, β-carotene, and sports and/or outdoor activities). Parent *et al*. reported an adjusted odds ratio of 1.04 [95% (classical) confidence interval: 0.49–2.22] for the association between night work and melanoma. The fact that the value 1 is within the classical confidence interval indicates that the association is not statistically significant. Again, the classical confidence interval itself, which spans all five regions, is also inconclusive.

Using the proposed method to reanalyze the data, the five-region test rejects H_I_ (a strong protective factor) and H_V_ (a strong risk factor) (p_I_ = 0.029 and p_V_ = 0.045, respectively). Similarly, the 95% five-region confidence interval is 0.50–2.00, excluding both the H_I_ and H_V_ regions. We see that while neither the classical test nor the classical confidence interval is conclusive about the risk of exposure, the five-region test (and the five-region confidence interval) at the same significance level of 0.05 can rule out the possibility that night work is a strong factor, risk or protective, for melanoma; it can only be one of the following three possibilities: a weak protective factor, no association, or a weak risk factor.

### Example 3. ‘A Weak Risk Factor’ vs. ‘A Risk Factor without Strength Information’

Li *et al*.^9^ recruited a total of 610 cases and 837 controls to examine the association between indoor air pollution from coal combustion and the risk of neural tube defects. Logistic regression was conducted adjusting for the matching variables (county of residence, season of conception, maternal ethnic group, and infant sex) and a number of other potential confounders (maternal age, education, multiparity, multiple births, history of pregnancy affected by birth defects, maternal influenza or fever, and passive smoking during the periconceptional period). Li *et al*. reported an adjusted odds ratio of 1.50 [95% (classical) confidence interval: 1.10–2.10] for the association between indoor air pollution from cooking and neural tube defects. The fact that the value 1 is not within the classical confidence interval indicates that the observed positive association between indoor air pollution from cooking and neural tube defects (OR = 1.50) is statistically significant. However, the classical confidence interval encompasses the H_IV_ (weak risk factor) and the H_V_ (strong risk factor) regions, so we cannot deduce the strength of this risk factor.

Using the proposed method to reanalyze the data, we find that except for the H_IV_ region (weak risk factor), all other 4 regions (p_I_ < 0.001, p_II_ = 0.014, p_III_ = 0.014, and p_V_ = 0.041, respectively) are rejected by the five-region test. The 95% five-region confidence interval is 1.10–2.00, which is fully embedded within the H_IV_ region. In this example, the classical method can only label indoor air pollution from cooking as a risk factor for neural tube defects. But the five-region method at the same significance level of 0.05 can be more specific in stating that it is a weak risk factor with an odds ratio no greater than 2.

## Discussion

The proposed five-region methods hinge on a proper demarcation of the five regions. For the odds ratio index used in this paper, the value ‘1’ was taken to be the center point, indicating no exposure-disease association. Therefore, we let a solitary {1} be a ‘region’ (H_III_). We then chose two cut-off points, an upper cut-off (c^upper^ > 1) and a lower one (c^lower^ < 1) which could have been values other than the ‘2’ and ‘0.5’ used in this paper, to mark the boundaries between strong and weak effects. We thus demarcated two regions (using c^upper^) for strong and weak risk factors (H_V_ and H_IV_) to the right of the H_III_ region, and to the left of that we demarcated two regions (using c^lower^) for strong and weak protective factors (H_I_ and H_II_). Ideally, if we let c^lower^ = 1/c^upper^, then the testing framework of the five-region test is completely symmetrical in the two directions of an exposure-disease association. By imposing symmetry, we avoid all appearance of prejudice on whether the study exposure is a risk or a protective factor. Also, note that the five-region demarcation should be done beforehand since a data-snooping demarcation will incur bias. The required sample sizes will be different with different demarcations of the five regions as well.

This paper uses the odds ratio index, which can be a crude odds ratio such as from a 2 × 2 table or an adjusted one such as from a logistic regression analysis. Liu *et al*.^10^ had previously also developed equivalence tests specifically for the odds ratio index. In fact, the five-region methods in this paper work for all indices, as long as a demarcation that respects the partitioning principle can be done.

In this paper, we invoked the normality assumption for the log odds ratio (large sample theory). For small sample sizes exact methods will be needed. One can calculate the four limits (one for the right one-sided, one for the left one-sided, and two for the two-sided) using the classical *exact* methods first. Next, a classical limit is adopted as one limit of the five-region confidence interval, if it matches the type of the test (one-sided or two-sided) that should be performed in the region it belongs to. We pick one (or both) cut-off point(s), as appropriate, for the limit(s), if the previous step cannot find one or more suitable limit. Finally, we examine the span of this *exact* five-region confidence interval to make conclusions for the *exact* five-region test. Here, the coverage probability of the exact five-region confidence interval is at least (1 − *α*) and the type I error rate of the exact five-region test is at most *α*. Web Appendix 5 provides the R code.

If a researcher opts for a more precise estimation, he/she will choose a method that produces a narrower confidence interval. In most circumstances, a five-region confidence interval (and its mathematical equivalent, the three-sided confidence interval) is at least as narrow as a classical one (Fig. 2). Only when the standard error is very small (sample size is very large) and the odds ratio estimate is either very large (in H_V_) or very small (in H_I_), can a five-region/three-sided confidence interval be wider than a classical one (Web Appendix 6). From this, we see that the five-region/three-sided methods trade the precision of an odds ratio far away out in the open regions for a better resolution when it is closer to the center (for differentiating whether the exposure is a risk factor, a protective factor, or neither, if the three-sided confidence interval is used to determine a five-region conclusion) or the two cut-off points (for differentiating whether its effect is strong or weak); positioning the c^upper^ and c^lower^ where an enhanced resolution is deemed most desirable is recommended. Finally, partitioning the parameter space of the (log) odds ratio can produce at most two open regions (where the precisions of the odds ratios can be traded off). The two open regions plus the remaining one closed region constitute a total of three ‘sides’, and thus no further performance improvement can be expected beyond Goeman *et al*.’s three-sided confidence interval^2^.

To characterize the association between an exposure and a disease, the current epidemiologic paradigm calls for a trio of inferential statistics to be presented for the odds ratio: a point estimate, a (classical) p-value, and a (classical) confidence interval, respectively. In this paper, we show that the five-region methods can describe a putative association more efficiently and informatively, including its presence or absence, as well as its direction and strength (if any association exists). The five-region methods are recommended for routine use during the analysis of epidemiologic data.

## Electronic supplementary material

Supplementary information.
