# Supplementary material for: A Five-Region Hypothesis Test for Exposure-Disease Associations
Source: Sci Rep. 2017 Jul 11;7:5131. doi: 10.1038/s41598-017-05301-4 (PMC5506070; doi:10.1038/s41598-017-05301-4)
Supplement: Supplementary file 1 — Supplementary information. [file 41598_2017_5301_MOESM1_ESM.pdf]

Web Appendices

accompanying the paper entitled

A Five-Region Hypothesis Test for Exposure-Disease Associations

by

Han-Yi Shih and Wen-Chung Lee

Web Appendix 1. A proof that a five-region test controls the family-wise error rate at α.

Web Appendix 2. A note showing that the five-region p-values correspond exactly to the five-region tests.

Web Appendix 3. R code for five-region test and confidence interval.

Web Appendix 4. R code for five-region sample size calculation.

Web Appendix 5. R code for exact five-region test and confidence interval.

Web Appendix 6. A comparison between the five-region confidence intervals and the classical confidence intervals, when the odds ratio estimates are in the open regions.

Web Appendix 1. A proof that a five-region test controls the family-wise error rate at α.

Let the event of a false rejection of the i th null hypothesis be denoted as F_i. We have, for $i = \mathrm{I}$, II,...,V, $\Pr(\mathrm{F}_i \mid \mathrm{OR} \in \mathrm{H}_i) \leq \alpha$ (because each of the five tests controls its respective type I error rate at α) and $\Pr(\mathrm{F}_j \mid \mathrm{OR} \in \mathrm{H}_i) = 0$ if $j \neq i$ (by the definition of a *false* rejection). Because the five regions are mutually exclusive, the true odds ratio must lie in one and only one region, say, the t th region. Therefore, the family-wise error rate is

$$\Pr\left(\bigcup_{i=1}^{5} \mathrm{F}_i \middle| \mathrm{OR} \in \mathrm{H}_t \right) \leq \sum_{i=1}^{5} \Pr(\mathrm{F}_i \mid \mathrm{OR} \in \mathrm{H}_t) = \Pr(\mathrm{F}_t \mid \mathrm{OR} \in \mathrm{H}_t) \leq \alpha.$$

Web Appendix 2. A note showing that the five-region p-values correspond exactly to the five-region tests.

p_I and p_V in (3) correspond to the one-sided p-values testing the cut-offs as specified in the H_I and H_V tests in (2), respectively. p_{III} in (3) corresponds to the classical two-sided p-value for association as specified in the H_{III} test in (2). p_{II} and p_{IV} in (3) are p-values corresponding to the 'two $\alpha/2$-level one-sided tests simultaneously' in (2), respectively. To be more exact, p_{II} in (3) uses a minimum of two one-sided p-values, one for the null hypothesis of $OR \geq 0.5$ and one for the null hypothesis of $OR < 1$, and multiply by 2. Thus, if either of these two one-sided p-values is less than or equal to $\alpha/2$, we have $p_{II} \leq \alpha$ and a rejection of H_{II} in (2). Similarly, p_{IV} in (3) uses a minimum of two one-sided p-values, one for the null hypothesis of $OR > 1$ and one for the null hypothesis of $OR \leq 2$, and multiply by 2. Thus, if either of these two one-sided p-values is less than or equal to $\alpha/2$, we have $p_{IV} \leq \alpha$ and a rejection of H_{IV} in (2).

Web Appendix 3. R code for five-region test and confidence interval.

```
################################################
#   Arguments for the function, five.region.test:          #
#     or: odds ratio estimate                              #
#     se: standard error of log odds ratio                 #
#     lc: lower cut-off point (default=0.5)                #
#     uc: upper cut-off point (default=2)                  #
#     alpha: significance level (default=0.05)             #
################################################


five.region.test=function( or, se, lc=0.5, uc=2, alpha=0.05 ){
  options(scipen=5)
  lor=log(or)
  lb=log(lc)
  ub=log(uc)
  if(lor<lb-qnorm(alpha,0,1)*se){
    newl=lor+qnorm(alpha,0,1)*se
  }else if(lb-qnorm(alpha,0,1)*se<=lor&lor<=lb-qnorm(alpha/2,0,1)*se){
    newl=lb
  }else if(lb-qnorm(alpha/2,0,1)*se<lor&lor<ub-qnorm(alpha/2,0,1)*se){
    newl=lor+qnorm(alpha/2,0,1)*se
  }else if(ub-qnorm(alpha/2,0,1)*se<=lor){
    newl=ub
  }
  if(lor<=lb-qnorm(1-alpha/2,0,1)*se){
    newu=lb
    }else if(lb-qnorm(1-alpha/2,0,1)*se<lor&lor<ub-qnorm(1-alpha/2,0,1)*se){
      newu=lor+qnorm(1-alpha/2,0,1)*se
    }else if(ub-qnorm(1-alpha/2,0,1)*se<=lor&lor<=ub-qnorm(1-alpha,0,1)*se){
      newu=ub
    }else if(ub-qnorm(1-alpha,0,1)*se<lor){
      newu=lor+qnorm(1-alpha,0,1)*se
  }
  z1=(lor-lb)/se
  z2=(lor-log(1))/se
```

```
z3=(lor-ub)/se
p1=1-pnorm(z1,0,1)
p21=pnorm(z1,0,1)
p22=1-pnorm(z2,0,1)
p2=2*min(p21,p22)
p3=2*(1-pnorm(abs(z2),0,1))
p41=pnorm(z2,0,1)
p42=1-pnorm(z3,0,1)
p4=2*min(p41,42)
p5=pnorm(z3,0,1)
p=round(c(p1,p2,p3,p4,p5),3)
for(i in 1:length(p)){
   if(p[i]<=0.0001) p[i]="<0.001"
}
new.p=matrix(p,5,1)
dimnames(new.p)=list(c("H1","H2","H3","H4","H5"),c("pvalue"))
r1=ifelse(z1>=qnorm(1-alpha,0,1),1,0)
r2=ifelse(z1<=qnorm(alpha/2,0,1)|z2>=qnorm(1-alpha/2,0,1),1,0)
r3=ifelse(abs(z2)>=qnorm(1-alpha/2,0,1),1,0)
r4=ifelse(z2<=qnorm(alpha/2,0,1)|z3>=qnorm(1-alpha/2,0,1),1,0)
r5=ifelse(z3<=qnorm(alpha,0,1),1,0)
rr1=ifelse(z1>=qnorm(0.95,0,1),"Rejected","                ")
rr2=ifelse(z1<=qnorm(0.025,0,1)|z2>=qnorm(0.975,0,1),"Rejected","                ")
rr3=ifelse(abs(z2)>=qnorm(0.975,0,1),"Rejected","                ")
rr4=ifelse(z2<=qnorm(0.025,0,1)|z3>=qnorm(0.975,0,1),"Rejected","                ")
rr5=ifelse(z3<=qnorm(0.05,0,1),"Rejected","                ")
new.r=rbind(rr1,rr2,rr3,rr4,rr5)
if(r1==0&r2==0&r3==0&r4==0&r5==0){
   con="Inconclusive"
}else if(r1==0&r2==1&r3==1&r4==1&r5==1){
   con="A Strong Protective Factor"
}else if(r1==1&r2==0&r3==1&r4==1&r5==1){
   con="A Weak Protective Factor"
}else if(r1==1&r2==1&r3==1&r4==0&r5==1){
   con="A Weak Risk Factor"
}else if(r1==1&r2==1&r3==1&r4==1&r5==0){
   con="A Strong Risk Factor"
```

```
    }else if(r1==0&r2==0&r3==1&r4==1&r5==1){
      con="A Protective Factor"
    }else if(r1==1&r2==1&r3==1&r4==0&r5==0){
      con="A Risk Factor"
    }else if(r1==1&r2==0&r3==0&r4==0&r5==0){
      con="Not a Strong Protective Factor"
    }else if(r1==0&r2==0&r3==0&r4==0&r5==1){
      con="Not a Strong Risk Factor"
    }else if(r1==1&r2==0&r3==0&r4==0&r5==1){
      con="Not a Strong Factor"
    }else{con="Error"
    }
    oldl=lor-qnorm(1-alpha/2,0,1)*se
    oldu=lor+qnorm(1-alpha/2,0,1)*se
    old.p=round(2*(1-pnorm(abs((lor-log(1))/se),0,1)),5)
    old=cbind(round(exp(oldl),3),round(exp(oldu),3),old.p)
    dimnames(old)=list(NULL,Estimate=c("CI lower","CI upper","pvalue"))
    new.ci=cbind(round(exp(newl),3),round(exp(newu),3))
    dimnames(new.ci)=list(NULL,Estimate=c("CI lower","CI upper"))
    new.rej=cbind(new.p,new.r)
    result=list("Classical"=old, "Five.Region.Test"=new.rej,
                "Five.Region.Confidence.Interval"=new.ci, "Conclusion"=con)
    return(result)
}

#Example 1
five.region.test(0.22,1.184)

#Example 2
five.region.test(1.04,0.385)

#Example 3
five.region.test(1.5,0.165)
```

Web Appendix 4. R code for five-region sample size calculation.

```
#################################################################################
#   Arguments for the function, five.region.sample.size:                        #
#     p: exposure prevalence                                                    #
#     OR: odds ratio                                                            #
#     con: specified conclusion (R: a risk factor; P: a protective factor;      #
#                                WR: a weak risk factor; WP: a weak protective factor;   #
#                                SR: a strong risk factor; SP: a strong protective factor;  #
#                                NSR: not a risk factor; NSP: not a strong protective factor;  #
#                                NSF: not a strong factor.)                     #
#     m: control-to-case ratio (default=1)                                      #
#     power.target: target power (default=0.80)                                 #
#     lc: lower cut-off point (default=0.5)                                     #
#     uc: upper cut-off point (default=2)                                       #
#     alpha: significance level (default=0.05)                                  #
#################################################################################

five.region.sample.size=function(p, OR, con, m=1, lc=0.5, uc=2, power.target=0.8, alpha=0.05){
  power=function(OR,n){
    rep=100000
    set.seed(1)
    n.control=n
    c=rbinom(rep,n.control,p)
    c[which(c==n.control)]=n.control-10^-8
    c[which(c==0)]=10^-8
    d=n.control-c
    p=OR*c/d
    pa=p/(1+p)
    n.case=ceiling(n/m)
    a=rbinom(rep,n.case,pa)
    a[which(a==n.case)]=n.case-10^-8
    a[which(a==0)]=10^-8
    b=n.case-a
    tables=cbind(a,b,c,d)
```

```
or=(a*d)/(b*c)
var=1/a+1/b+1/c+1/d
se=sqrt(var)
lor=log(or)
lb=log(lc)
ub=log(uc)
newl=c()
newu=c()
oldl=c()
oldu=c()
new.no=c()
old.no=c()
for(i in 1:rep){
  if(lor[i]<lb-qnorm(alpha,0,1)*se[i]){
     newl[i]=lor[i]+qnorm(alpha,0,1)*se[i]
  }else if(lb-qnorm(alpha,0,1)*se[i]<=lor[i]&lor[i]<=lb-qnorm(alpha/2,0,1)*se[i]){
     newl[i]=lb
  }else if(lb-qnorm(alpha/2,0,1)*se[i]<lor[i]&lor[i]<ub-qnorm(alpha/2,0,1)*se[i]){
     newl[i]=lor[i]+qnorm(alpha/2,0,1)*se[i]
  }else if(ub-qnorm(alpha/2,0,1)*se[i]<=lor[i]){
     newl[i]=ub
  }
  if(lor[i]<=lb-qnorm(1-alpha/2,0,1)*se[i]){
     newu[i]=lb
  }else if(lb-qnorm(1-alpha/2,0,1)*se[i]<lor[i]&lor[i]<ub-qnorm(1-alpha/2,0,1)*se[i]){
     newu[i]=lor[i]+qnorm(1-alpha/2,0,1)*se[i]
  }else if(ub-qnorm(1-alpha/2,0,1)*se[i]<=lor[i]&lor[i]<=ub-qnorm(1-alpha,0,1)*se[i]){
     newu[i]=ub
  }else if(ub-qnorm(1-alpha,0,1)*se[i]<lor[i]){
     newu[i]=lor[i]+qnorm(1-alpha,0,1)*se[i]
  }
  oldl[i]=lor[i]-qnorm(1-alpha/2,0,1)*se[i]
  oldu[i]=lor[i]+qnorm(1-alpha/2,0,1)*se[i]
  if(con=="R"){
     new.no[i]=ifelse(0<newl[i],1,0)
     old.no[i]=ifelse(0<oldl[i],1,0)
  }else if(con=="P"){
```

```
        new.no[i]=ifelse(newu[i]<0,1,0)
        old.no[i]=ifelse(oldu[i]<0,1,0)
    }else if(con=="WR"){
        new.no[i]=ifelse(0<newl[i]&&newu[i]<=ub,1,0)
        old.no[i]=ifelse(0<oldl[i]&&oldu[i]<=ub,1,0)
    }else if(con=="WP"){
        new.no[i]=ifelse(lb<=newl[i]&&newu[i]<0,1,0)
        old.no[i]=ifelse(lb<=oldl[i]&&oldu[i]<0,1,0)
    }else if(con=="SR"){
        new.no[i]=ifelse(ub<=newl[i],1,0)
        old.no[i]=ifelse(ub<=oldl[i],1,0)
    }else if(con=="SP"){
        new.no[i]=ifelse(newu[i]<=lb,1,0)
        old.no[i]=ifelse(oldu[i]<=lb,1,0)
    }else if(con=="NSR"){
        new.no[i]=ifelse(newu[i]<=ub,1,0)
        old.no[i]=ifelse(oldu[i]<=ub,1,0)
    }else if(con=="NSP"){
        new.no[i]=ifelse(lb<=newl[i],1,0)
        old.no[i]=ifelse(lb<=oldl[i],1,0)
    }else if(con=="NSF"){
        new.no[i]=ifelse(lb<=newl[i]&&newu[i]<=ub,1,0)
        old.no[i]=ifelse(lb<=oldl[i]&&oldu[i]<=ub,1,0)
    }
   }
   new.power=mean(new.no)
   old.power=mean(old.no)
   return(cbind(new.power,old.power))
}
bisection=function(OR,r){
    l=0
    u=20000
    v=u
    p=power(OR,v)[r]
    for(i in 2:50){
        if(p<power.target){
              l=v
```

```
            u=u
        }else if(p>power.target){
            l=l
            u=v
        }else if(p==power.target){
            break
        }
        v=(l+u)/2
        p=power(OR,round(v))[r]
      }
      return(v)
    }
    s=matrix(NA,length(OR),3)
    for(i in 1:length(OR)){
      s[i,1]=OR[i]
      s[i,2]=ceiling(bisection(OR[i],1)*(1+1/m))
      s[i,3]=ceiling(bisection(OR[i],2)*(1+1/m))
    }
    colnames(s)=list("OR","Five Region","Classical")
    return(s)
}

#Examples (when OR=1.5)
five.region.sample.size (0.4,1.5,"NSP")
five.region.sample.size (0.4,1.5,"R")
five.region.sample.size (0.4,1.5,"NSF")
five.region.sample.size (0.4,1.5,"WR")

#Examples (when OR=2.5)
five.region.sample.size (0.4,2.5,"NSP")
five.region.sample.size (0.4,2.5,"R")
five.region.sample.size (0.4,2.5,"SR")
```

Web Appendix 5. R code for exact five-region test and confidence interval.

```
#############################################################################
#   Arguments for the function, five.region.exact:                          #
#     lower.one: exact lower limit for classical one-sided confidence interval      #
#     upper.one: exact upper limit for classical one-sided confidence interval      #
#     lower.two: exact lower limit for classical two-sided confidence interval      #
#     upper.two: exact upper limit for classical two-sided confidence interval      #
#     lc: lower cut-off point (default=0.5)                                 #
#     uc: upper cut-off point (default=2)                                   #
#############################################################################

five.region.exact=function(lower.one, upper.one, lower.two, upper.two, lc=0.5, uc=2){
  if(lower.one<lc){
    newl=lower.one
  }else if(lc<lower.one&lower.two<lc){
    newl=lc
  }else if(lc<lower.two&lower.two<uc){
    newl=lower.two
  }else if(uc<lower.two){
    newl=uc
  }
  if(uc<upper.one){
    newu=upper.one
  }else if(upper.one<uc&uc<upper.two){
    newu=uc
  }else if(lc<upper.two&upper.two<uc){
    newu=upper.two
  }else if(upper.two<lc){
    newu=lc
  }
  if(newl<lc&uc<newu){
    con="Inconclusive"
    H1="          "
    H2="          "
    H3="          "
```

```
    H4="                "
    H5="                "
}else if(newl<lc&newu<=lc){
    con="A Strong Protective Factor"
    H1="                "
    H2="Rejected"
    H3="Rejected"
    H4="Rejected"
    H5="Rejected"
}else if(lc<=newl&newu<1){
    con="A Weak Protective Factor"
    H1="Rejected"
    H2="                "
    H3="Rejected"
    H4="Rejected"
    H5="Rejected"
}else if(1<newl&newu<=uc){
    con="A Weak Risk Factor"
    H1="Rejected"
    H2="Rejected"
    H3="Rejected"
    H4="                "
    H5="Rejected"
}else if(uc<=newl&uc<newu){
    con="A Strong Risk Factor"
    H1="Rejected"
    H2="Rejected"
    H3="Rejected"
    H4="Rejected"
    H5="                "
}else if(newl<lc&newu<1){
    con="A Protective Factor"
    H1="                "
    H2="                "
    H3="Rejected"
    H4="Rejected"
    H5="Rejected"
```

```
    }else if(1<newl&uc<newu){
      con="A Risk Factor"
      H1="              "
      H2="              "
      H3="              "
      H4="Rejected"
      H5="Rejected"
    }else if(lc<=newl&uc<newu){
      con="Not a Strong Protective Factor"
      H1="Rejected"
      H2="              "
      H3="              "
      H4="              "
      H5="              "
    }else if(newl<lc&newu<=uc){
      con="Not a Strong Risk Factor"
      H1="              "
      H2="              "
      H3="              "
      H4="              "
      H5="Rejected"
    }else if(lc<=newl&newu<=uc){
      con="Not a Strong Factor"
      H1="Rejected"
      H2="              "
      H3="              "
      H4="              "
      H5="Rejected"
    }else{con="Error"
    }
    ci=cbind(newl,newu)
    colnames(ci)=list("lower","upper")
    r=rbind(H1,H2,H3,H4,H5)
    result=list("Exact.Confidence.Interval"=ci,r,"Conclusion"=con)
    return(result)
}
```

Web Appendix 6. A comparison between the five-region confidence intervals (solid lines, with a solid circle at one limit indicating inclusiveness, a hollow circle, exclusiveness) and the classical confidence intervals (dashed lines), when the odds ratio estimates are in the open regions (left panels: SE=0.60; middle panels: SE=0.42; right panels: SE=0.18).

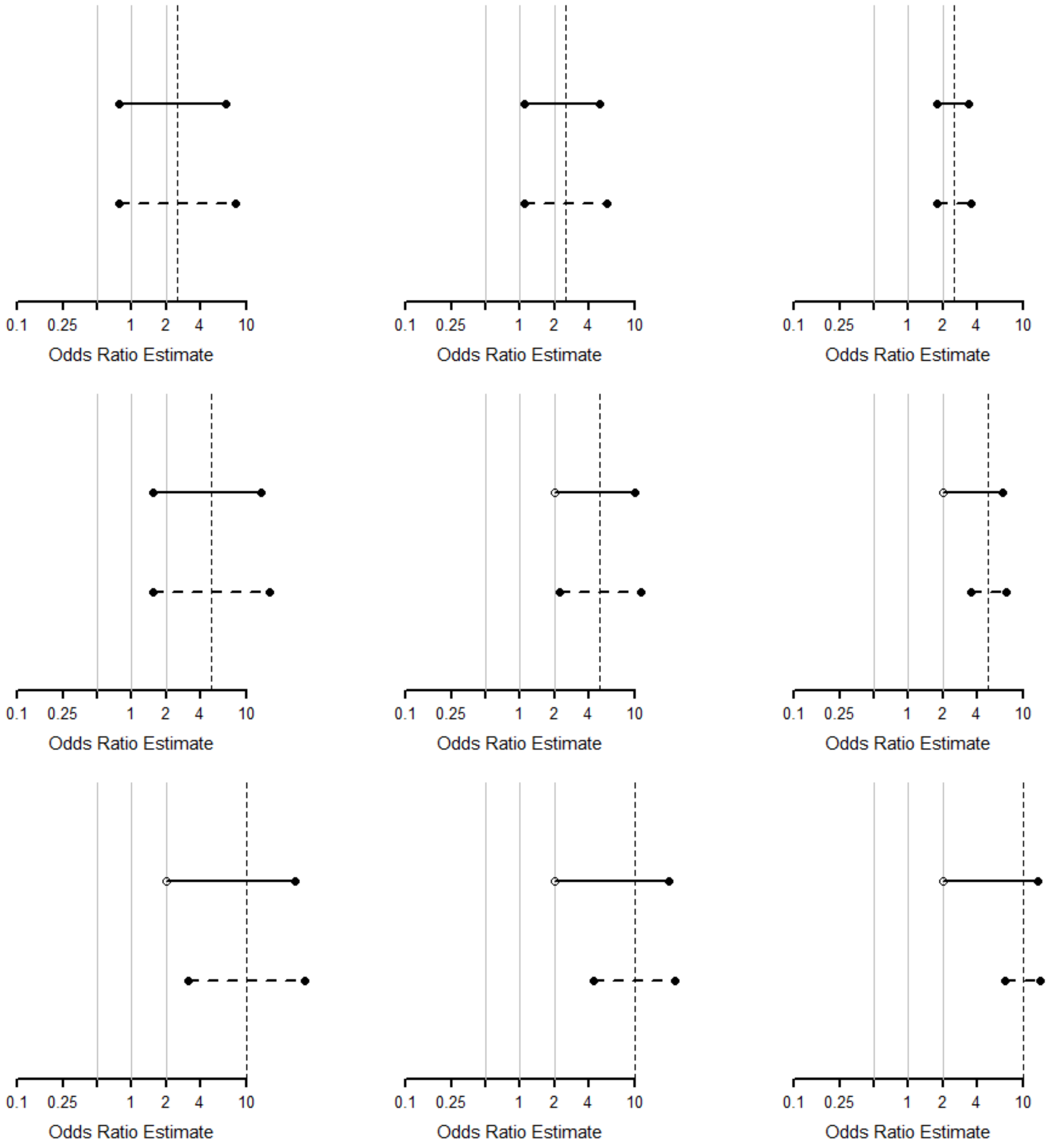
0.1 0.25 1 2 4 10
Odds Ratio Estimate
0.1 0.25 1 2 4 10
Odds Ratio Estimate
0.1 0.25 1 2 4 10
Odds Ratio Estimate
0.1 0.25 1 2 4 10
Odds Ratio Estimate
0.1 0.25 1 2 4 10
Odds Ratio Estimate
0.1 0.25 1 2 4 10
Odds Ratio Estimate
0.1 0.25 1 2 4 10
Odds Ratio Estimate
0.1 0.25 1 2 4 10
Odds Ratio Estimate
0.1 0.25 1 2 4 10
Odds Ratio Estimate

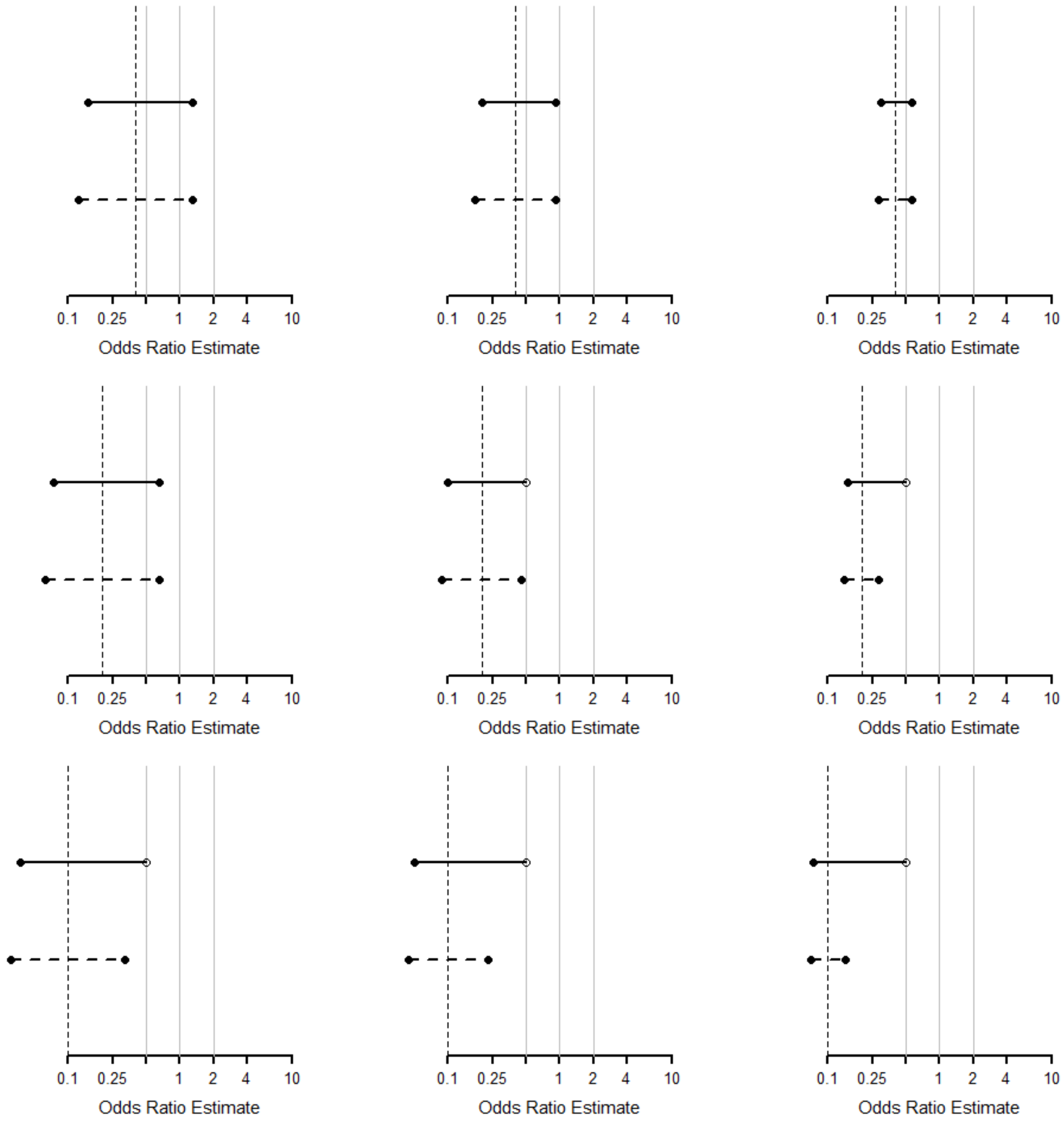
0.1 0.25 1 2 4 10
Odds Ratio Estimate
0.1 0.25 1 2 4 10
Odds Ratio Estimate
0.1 0.25 1 2 4 10
Odds Ratio Estimate
0.1 0.25 1 2 4 10
Odds Ratio Estimate
0.1 0.25 1 2 4 10
Odds Ratio Estimate
0.1 0.25 1 2 4 10
Odds Ratio Estimate
0.1 0.25 1 2 4 10
Odds Ratio Estimate
0.1 0.25 1 2 4 10
Odds Ratio Estimate
0.1 0.25 1 2 4 10
Odds Ratio Estimate
